# P-812. Not Little Adults: Key Differences in Antimicrobial Resistance in Australian Bloodstream Isolates between Children and Adults, 2020-2021

**DOI:** 10.1093/ofid/ofae631.1004

**Published:** 2025-01-29

**Authors:** Anita Williams, Geoff W Coombs, Jan Bell, Denise A Daley, Shakeel Mowlaboccus, Penelope A Bryant, Anita Jane Campbell, Louise Cooley, Annaleise Howard-Jones, Jon Iredell, Adam D Irwin, Brendan McMullan, Morgyn Warner, Phoebe Williams, Christopher C Blyth

**Affiliations:** Telethon Kids Institute, Perth, Western Australia, Australia; Antimicrobial Resistance and Infectious Diseases (AMRID) Research Laboratory, Murdoch University, Perth, Western Australia, Australia; Australian Group on Antimicrobial Resistance, Adelaide, South Australia, Australia; Department of Microbiology, PathWest Laboratory Medicine WA, Fiona Stanley Hospital, Perth, Western Australia, Australia; Antimicrobial Resistance and Infectious Diseases (AMRID) Research Laboratory, Murdoch University, Perth, Western Australia, Australia; Infectious Diseases, The Royal Children's Hospital, Melbourne, Victoria, Australia; Perth Children's Hospital, Telethon Kids Institute Wesfarmers Centre of Vaccines and Infectious Diseases, Nedlands, Australia; Department of Infectious Diseases, Royal Hobart Hospital, Hobart, Tasmania, Australia; NSW Health Pathology, Sydney, Alabama; Sydney Institute for Infectious Diseases (SydneyID), The University of Sydney, Sydney, New South Wales, Australia; UQ Centre for Clinical Research, The University of Queensland, Brisbane, Queensland, Australia; Sydney Children's Hospital Randwick, New South Wales, Australia, Sydney, New South Wales, Australia; The Queen Elizabeth Hospital, Central Adelaide Health Network, Adelaide, South Australia, Australia; Department of Immunology and Infectious Diseases, Sydney Children’s Hospital, Sydney, New South Wales, Australia; Wesfarmers Centre of Vaccines and Infectious Diseases, Telethon Kids Institute, Nedlands, Western Australia, Australia

## Abstract

**Background:**

The incidence, risks and organisms causing bloodstream infections (BSI) differ between children and adults due to distinct comorbidities, procedures and antibiotic exposures. Age-specific data are required to inform targeted interventions, empiric treatment and guideline development. We aimed to compare the incidence, risk factors and resistance patterns of bacteria causing BSI in children and adults (2020-21).

**Methods:**

The Australian Group on Antimicrobial Resistance (AGAR) is a national hospital-based BSI surveillance program reporting on *Staphylococcus aureus, Enterococcus* spp. and key gram-negative pathogens.

**Results:**

Data from 25,958 isolates were assessed (children: 1,679; adults: 24,279). The most common organisms in children and adults were *Escherichia coli* (20.9 vs 39.1%) and *S. aureus* (36.2 vs 20.9%). *E. faecalis* and non-typhoidal *Salmonella* spp. were more frequent in children (7.3% and 4.4% of surveyed organisms in children vs 5.1% and 0.5% in adults). BSI were more often community onset (69.0% children; 76.4% adults). 30-day mortality was significantly lower in children (3.3% vs 9.8%).

Enterobacterales resistance was more common in children: e.g. to gentamicin/tobramycin (11.6% of child isolates; 8.5%, adult isolates; rate ratio [RR]: 1.4 [95%CI: 1.1-1.7]) and piperacillin-tazobactam resistance (11.2%; 8.6%; RR: 1.3 [1.0-1.6]). However, there was no difference observed in Enterobacterales resistance to cephalosporins, ciprofloxacin, meropenem or multi-drug resistance status. AMR prevalence in *Pseudomonas* and *Acinetobacter* spp isolates were also similar between children and adults.

Methicillin-resistant *S. aureus* isolates were less common in children (13.2 vs 17.7%; RR: 0.7 [0.6-0.9]). Rates of clindamycin and cotrimoxazole resistance in *S. aureus* were similar to adults.

Children had a lower proportion of *E. faecium* infections (24.1 vs 39.8%) and the rate of vancomycin-resistant *E. faecium* in children was half that in adults (19.5 vs 37.2%; RR; 0.5, [0.2-1.0]).

**Conclusion:**

Analysis of national AMR data identifies unique trends in children. Ongoing clinical surveillance, targeted prevention, antimicrobial stewardship strategies and research to evaluate AMR drivers in children are required.

**Disclosures:**

**All Authors**: No reported disclosures

